# CD8^+^ TEMRAs in severe asthma associate with asthma symptom duration and escape proliferation arrest

**DOI:** 10.1172/jci.insight.185061

**Published:** 2025-03-06

**Authors:** Richard P. Ramonell, Timothy B. Oriss, Jessica C. McCreary-Partyka, Sagar L. Kale, Nicole R. Brandon, Mark A. Ross, Marc C. Gauthier, Molin Yue, Taylor J. Nee, Sudipta Das, Wei Chen, Alok V. Joglekar, Prabir Ray, Claudette M. St Croix, Dhivyaa Rajasundaram, Sally E. Wenzel, Anuradha Ray

**Affiliations:** 1 Department of Medicine, Division of Pulmonary, Allergy and Critical Care Medicine,; 2 Asthma and Environmental Lung Health Institute at UPMC,; 3 Department of Immunology,; 4 Department of Cell Biology,; 5 Center for Biological Imaging,; 6 Department of Pediatrics,; 7 Center for Systems Immunology, and; 8 Department of Environmental and Occupational Health, University of Pittsburgh, Pittsburgh, Pennsylvania.

**Keywords:** Immunology, Pulmonology, Asthma, Cytokines, T cells

## Abstract

Aberrant immune response is a hallmark of asthma, with 5%–10% of patients suffering from severe disease exhibiting poor response to standard treatment. A better understanding of the immune responses contributing to disease heterogeneity is critical for improving asthma management. T cells are major players in the orchestration of asthma, in both mild and severe disease, but it is unclear whether specific T cell subsets influence asthma symptom duration. Here we show a significant association of airway CD8^+^ effector memory T cells re-expressing CD45RA (TEMRAs), but not CD8^+^CD45RO^+^ or tissue-resident memory T cells, with asthma duration in patients with severe asthma (SA) but not mild to moderate asthma (MMA). Higher frequencies of IFN-γ^+^CD8^+^ TEMRAs compared with IFN-γ^+^CD45RO^+^ T cells were detected in SA airways, and the TEMRAs from patients with SA but not MMA proliferated ex vivo, although both expressed cellular senescence-associated biomarkers. Prompted by the transcriptomic profile of SA CD8^+^ TEMRAs and proliferative response to IL-15, airway *IL15* expression was higher in patients with SA compared with MMA. Additionally, *IL15* expression in asthmatic airways negatively correlated with lung function. Our findings add what we believe is a new dimension to understanding asthma heterogeneity, identifying IL-15 as a potential target for treatment.

## Abstract 2

Our study describes a significant association of airway CD8+ TEMRA cells with asthma duration in patients with severe asthma but not mild to moderate asthma identifying a novel target for asthma management in treatment-refractory asthma.

## Graphical abstract

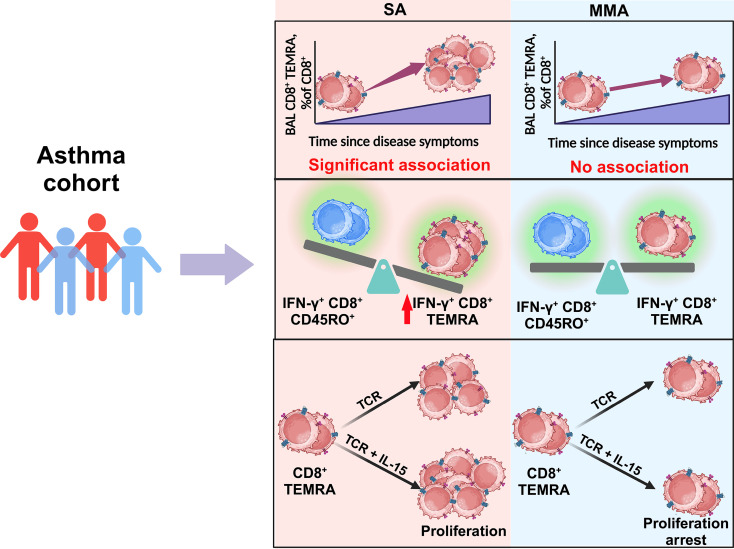

## Introduction

Asthma is a chronic heterogeneous disease of the airways affecting more than 300 million people worldwide and is clinically characterized by variable respiratory symptoms and reversible airflow limitation on pulmonary function testing (1–4). The vast majority of patients have mild to moderate asthma (MMA) but an estimated 5%–10% have a clinical diagnosis of severe asthma (SA) (1, 2, 4–7). Disease symptoms in patients with SA are characteristically poorly managed by the current standard of care, inhaled corticosteroids (CS), even when used at high doses or accompanied by oral CS (2, 4, 6, 7).

Since the early 1990s, immunophenotyping of asthma in humans and animal models has established an important role of Th2/type 2 (T2) cells as mediators of asthma pathogenesis, in both MMA and SA (2, 7–9). However, use of more advanced tools to study immune cells at the single-cell level in combination with machine learning showed activation of additional immune pathways that include type 1 (T1) T cells, and in some patients, also Th17 cells, in the setting of more severe disease (6, 10–13). These pathways are unaffected by T2-directed biologics and recalcitrant to CS, identifying an unmet need for new avenues for managing disease in these patients.

T lymphocytes in chronic age-associated illnesses are known to acquire highly differentiated phenotypes and become senescent, defined as cells in permanent cell-cycle arrest (14). While replicative senescence is typically associated with old age (15, 16), there is increasing appreciation for stress-induced premature senescence in lung diseases such as idiopathic pulmonary fibrosis and chronic obstructive pulmonary disease (17, 18). The senescent phenotype is hypothesized to exist to prevent the progeny of damaged cells from undergoing malignant conversion, yet senescent cells, including T cells, can be highly inflammatory, acquiring a senescence-associated secretory phenotype (SASP), when the cells secrete a range of biomolecules, including proinflammatory cytokines, such as IFN-γ and TNF-α, chemokines, matrix-associated metalloproteinases, and bioactive lipids, among others (19–22). Multiple memory T cell subsets infiltrate the airways of asthmatics, as we and others have shown (6, 12), which begs the question whether one or more of these cell types associate with the time since the onset of asthma symptoms. One subset of memory T cells, effector memory T cells re-expressing CD45RA (TEMRAs), are terminally differentiated T cells that lose cell surface expression of CD27, CD28, and CD127 but regain expression of CD45RA, the latter typically expressed by naive T cells (23, 24). Both CD4^+^ and CD8^+^ TEMRAs have been described in humans but these cells are not present in mice. TEMRAs have been associated with multiple chronic inflammatory diseases that include autoimmune disease (25) and Alzheimer disease (26). While TEMRA abundance is commonly associated with chronological age (15), the blood, spleen, and lung are particularly prone to harboring CD8^+^ TEMRAs regardless of age (24). Emerging literature emphasizes roles for persistent viral infections and inflammatory disease conditions as drivers of TEMRAs, especially CD8^+^ TEMRAs, with the cells showing features of cellular senescence (15, 27, 28). Although the inflammatory characteristics of CD8^+^ TEMRAs should enable their clearance by the immune system, evidence suggests that age, persistent viral infections, and disease-associated immune dysfunction can cause their accumulation (15, 27, 28). A consequence of the increased numbers of senescent CD8^+^ TEMRAs with enhanced innate-like functions, typically associated with natural killer (NK) cells (29), is induction of chronic sterile inflammation that impairs tissue homeostasis and causes tissue damage, a state collectively termed inflammaging (15, 30).

Our earlier studies of immune and molecular mechanisms of SA involving analysis of airway transcriptomic data (31, 32), airway immune cells in humans and in mouse models (10, 33–36), and more recent studies of bronchoalveolar lavage (BAL) and airway epithelial cells utilizing advanced bioinformatic tools (6, 11) highlight the presence of an increased T1/IFN-γ immune response in a subset of these patients who poorly respond to CS. This T1^hi^ steroid-resistant phenotype has been also appreciated by others in both adults (12, 37) and children (38, 39). Notably, the combined presence of T1 and T2 immune responses appears to be a hallmark of the sickest of asthma patients (31, 35, 37), with IFN-γ or the T1 phenotype negatively associating with airway obstruction, as measured by the percentage of predicted forced expiratory volume in 1 second (FEV1), a measure of lung function (10, 40). Because of the association of TEMRAs with viral infections, which are features of steroid-resistant SA in both children (38) and adults (37), and their propensity for IFN-γ secretion, TEMRAs are also a potential source of IFN-γ in SA in addition to effector/memory and tissue-resident memory (TRM) T cells (6, 10, 33, 35).

Here, we examined TEMRAs in patients with a diagnosis of asthma. We found an increase in the percentage of CD8^+^, but not CD4^+^, TEMRAs in the peripheral blood (PB) and BAL cells of patients with SA, but not MMA, positively correlating with asthma symptom duration. Compared with other memory T cells in patients with SA, the percentages of cells expressing IFN-γ and senescence-associated biomarkers were higher in CD8^+^ TEMRAs. Patients with SA were found to have higher airway expression of *IL15*, encoding a known TEMRA activator and chemokine for CD8^+^ T cells (41, 42). *IL15* expression positively correlated with expression of T cell–associated genes and *IFNG* but negatively correlated with lung function. However, as opposed to canonical descriptions of TEMRAs and senescence, CD8^+^ TEMRAs were more proliferative in patients with SA than MMA in response to T cell receptor (TCR) stimulation and IL-15, suggesting a potential mechanism for immunopathology in SA.

## Results

### CD8^+^ TEMRAs are detectable in PB and BAL cells in patients with asthma.

**Figure 1:**
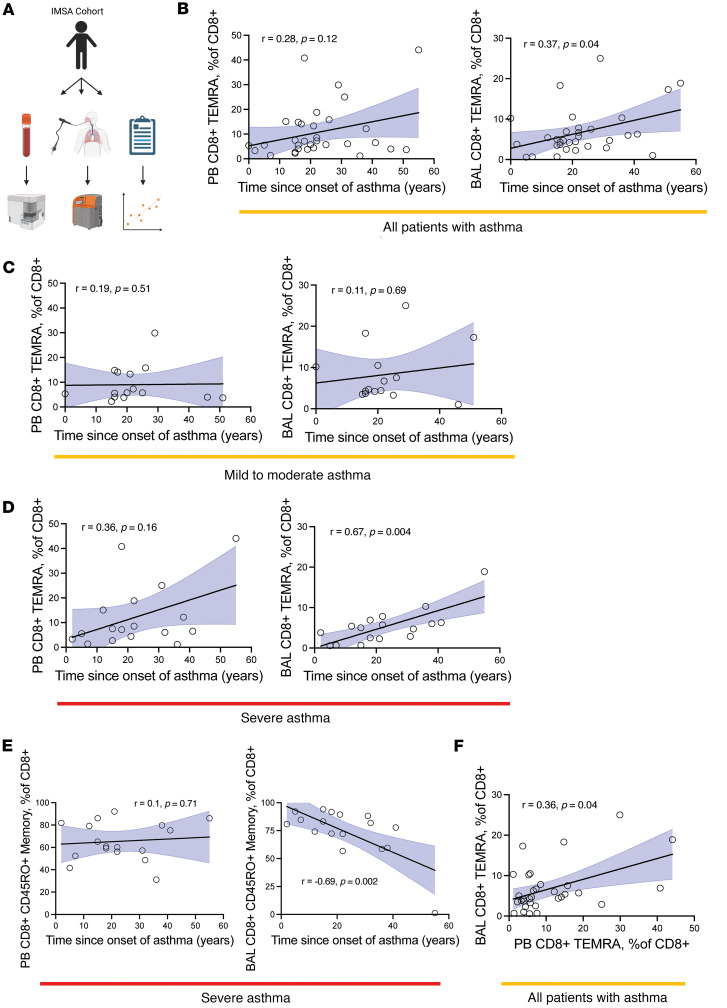
Association of T cell subsets with time since the onset of asthma symptoms. (**A**) Overall experimental design. (**B**) Linear regression of PB CD8^+^ TEMRAs (percentage of CD8^+^ cells) as a function of the time in years since the onset of an individual’s asthma symptoms. (**C**) Linear regression of PB (left) and BAL (right) CD8^+^ TEMRAs (percentage of CD8^+^ cells) from patients with MMA as a function of the time in years since the onset of an individual’s asthma symptoms. (**D**) Linear regression of PB (left) and BAL (right) CD8^+^ TEMRAs (percentage of CD8^+^ cells) from patients with SA as a function of the time in years since the onset of a subject’s asthma symptoms. (**E**) Linear regression of PB (left) and BAL (right) CD8^+^CD45RO^+^ memory (percentage of CD8^+^ cells) from patients with SA as a function of the time in years since the onset of a subject’s asthma symptoms. (**F**) Linear regression of PB CD8^+^ TEMRAs (percentage of CD8^+^ cells) as a function of BAL CD8^+^ TEMRAs (percentage of CD8^+^ cells). Statistical significance in **B**–**F** determined using Spearman’s nonparametric correlations, with solid black lines representing simple linear regression line and shaded blue area representing 95% confidence interval.

PB mononuclear cells (PBMCs) from patients with MMA or SA were stained with a high-dimensional flow cytometry panel of antibodies and analyzed in combination with clinical metadata obtained from the Immune Mechanisms in Severe Asthma (IMSA) study (see Methods). These data were paired with previously generated mass cytometry (CyTOF) data (Figure 1A) (6) from BAL cells that were manually reanalyzed to investigate T cell subsets. Demographic and clinical characteristics of donors can be found in Supplemental Table 1; supplemental material available online with this article; https://doi.org/10.1172/jci.insight.185061DS1 Using a gating strategy that accounted for the differences in T cell subsets in PB and BAL cells (Supplemental Figure 1), we measured the prevalence of CD4^+^ and CD8^+^ TEMRAs in these 2 compartments in patients with MMA and SA.

CD4^+^ TEMRAs were low in abundance in both PB and BAL and were therefore not examined further. Based on the association of TEMRAs with age, we hypothesized that CD8^+^ TEMRAs would increase in frequency with age but discovered no relationship between the percentage of CD8^+^ TEMRAs in PB or BAL and donor age. However, we found a positive correlation between percentage CD8^+^ TEMRAs in the CD8^+^ T cell compartment and the time since the onset of asthma symptoms in both the PB and BAL (Figure 1B) after combining all asthma patients (*n* = 32). When examined by asthma severity, this trend was not observed in percentage CD8^+^ TEMRAs from patients with MMA in either compartment (Figure 1C), but in patients with SA, there was a positive association with CD8^+^ TEMRAs in PB that reached statistical significance in BAL (Figure 1D). Interestingly, there was no association between the time since the onset of asthma symptoms and CD8^+^CD45RO^+^ memory T cells (a composite of effector memory T cells and central memory T cells) in PB and there was a significant negative association with these cells in BAL (Figure 1E) among patients with SA. These trends remained when evaluating all patients with asthma (MMA and SA combined) (Supplemental Figure 2A). We previously reported the presence of CD8^+^ TRM T cells in the airways of patients with SA (6). However, we did not detect any association between CD8^+^ TRMs and the time since onset of symptoms in patients with either MMA or SA (Supplemental Figure 2B).

Taken together, these data suggested that CD8^+^ TEMRAs increasingly comprise more of the CD8^+^ compartment in patients with SA, but not MMA, the longer the individual has had asthma. We found a significant positive relationship between CD8^+^ TEMRAs in PB and BAL in all patients with asthma (Figure 1F), suggesting that TEMRAs accumulate at similar rates in BAL and PB and that circulating PB CD8^+^ TEMRAs may reflect BAL CD8^+^ TEMRA biology. This is in accordance with prior studies demonstrating the presence of CD8^+^ TEMRAs in the lung and PB (24).

### PB CD8^+^ TEMRAs in patients with SA express T1 cytokines and show senescent features.

**Figure 2:**
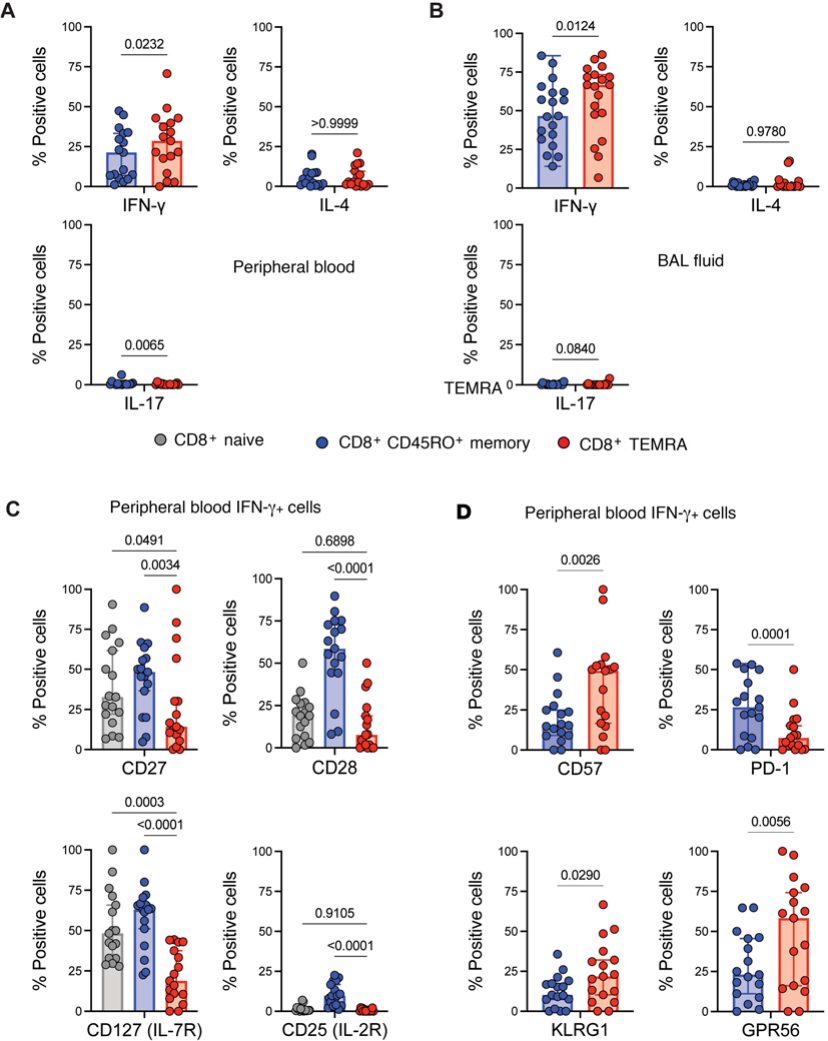
Compared with CD8^+^CD45RO^+^ memory T cells, CD8^+^ TEMRAs in PB display higher percentages of IFN-γ^+^ and senescence marker–expressing cells. (**A**) Percentage of cytokine-expressing PB cells from patients with SA. (**B**) Percentages of BAL cells expressing cytokines from patients with SA. (**C** and **D**) Percentages of PB IFN-γ^+^ cells expressing senescence-associated surface proteins from patients with SA. Statistical significance for data in **A**, **B**, and **D** determined using Wilcoxon’s matched-pairs signed rank test and for data in **C** determined using Kruskal-Wallis followed by multiple pairwise comparisons. Data represent median ± 95% confidence interval.

CD8^+^ TEMRAs in other chronic illnesses have been described as senescent, hyperinflammatory, and terminally differentiated (26, 42–45). These cells express a marker of naive T cells, CD45RA, yet do not retain other hallmarks of naive T cells and are skewed toward T1 cytokine secretion (42, 43, 45). We therefore hypothesized that PB CD8^+^ TEMRAs would be similar to TEMRAs in other human illnesses, would express biomarkers associated with senescence at higher rates in patients with SA, and would exhibit a SASP with more IFN-γ expression. To address this, we measured intracellular expression of cytokines canonically associated with T1, T2, and T17 immune programs. Whether in PB (Figure 2A) or BAL cells (Figure 2B), very low percentages of CD8^+^ TEMRAs or CD45RO^+^ T cells expressing either IL-4 or IL-17 were detected. However, a significantly higher percentage of CD8^+^ TEMRAs expressed IFN-γ when compared with CD8^+^CD45RO^+^ memory T cells, especially in the BAL (Figure 2B). We did not detect a significant difference between IFN-γ^+^CD8^+^ TEMRAs and IFN-γ^+^CD8^+^CD45RO^+^ memory T cells in the BAL cells of patients with MMA (Supplemental Figure 2C). We therefore gated on the IFN-γ^+^ cell subset in T cells from patients with SA for further analysis and found lower percentages of CD8^+^ TEMRAs with markers of activation, including CD27 and CD28 when compared with other subsets (Figure 2C), consistent with prior TEMRA descriptions (26, 43). Lower percentages of CD8^+^ TEMRAs expressed IL-2R (CD25) and IL-7R (CD127) than CD8^+^CD45RO^+^ memory T cells (Figure 2C). In addition, a smaller percentage of PB CD8^+^IFN-γ^+^ TEMRAs in SA expressed PD-1 when compared with CD8^+^IFN-γ^+^CD45RO^+^ memory T cells, showing they did not display complete or intermediate levels of exhaustion (46), and a significantly higher percentage expressed a biomarker of senescence, CD57 (Figure 2D) (29). Compared with CD8^+^IFN-γ^+^CD45RO^+^ T cells, higher percentages of CD8^+^IFN-γ^+^ TEMRAs expressed other senescence-associated markers such as killer cell lectin-like G1 (KLRG1) (29) and adhesion G protein–coupled receptor 56 (GPR56) (Figure 2D) (47). Notably, expression of these biomarkers of T cell senescence was also detected in the PB of CD8^+^IFN-γ^+^ TEMRAs in patients with MMA (Supplemental Figure 2D).

These data supported our hypothesis that CD8^+^ TEMRAs in patients with SA recapitulate the hyperinflammatory, senescent phenotype that has been documented in other chronic inflammatory states in humans such as organ transplantation (42), chronic infection (28), and autoimmune disease (25). To the best of our knowledge, CD8^+^ TEMRAs have not been described in asthma previously and yet could be a significant source of T1 cytokines in subsets of patients with SA.

### PB CD8^+^ TEMRAs show significant transcriptional divergence from CD8^+^CD45RO^+^ memory T cells.

**Figure 3:**
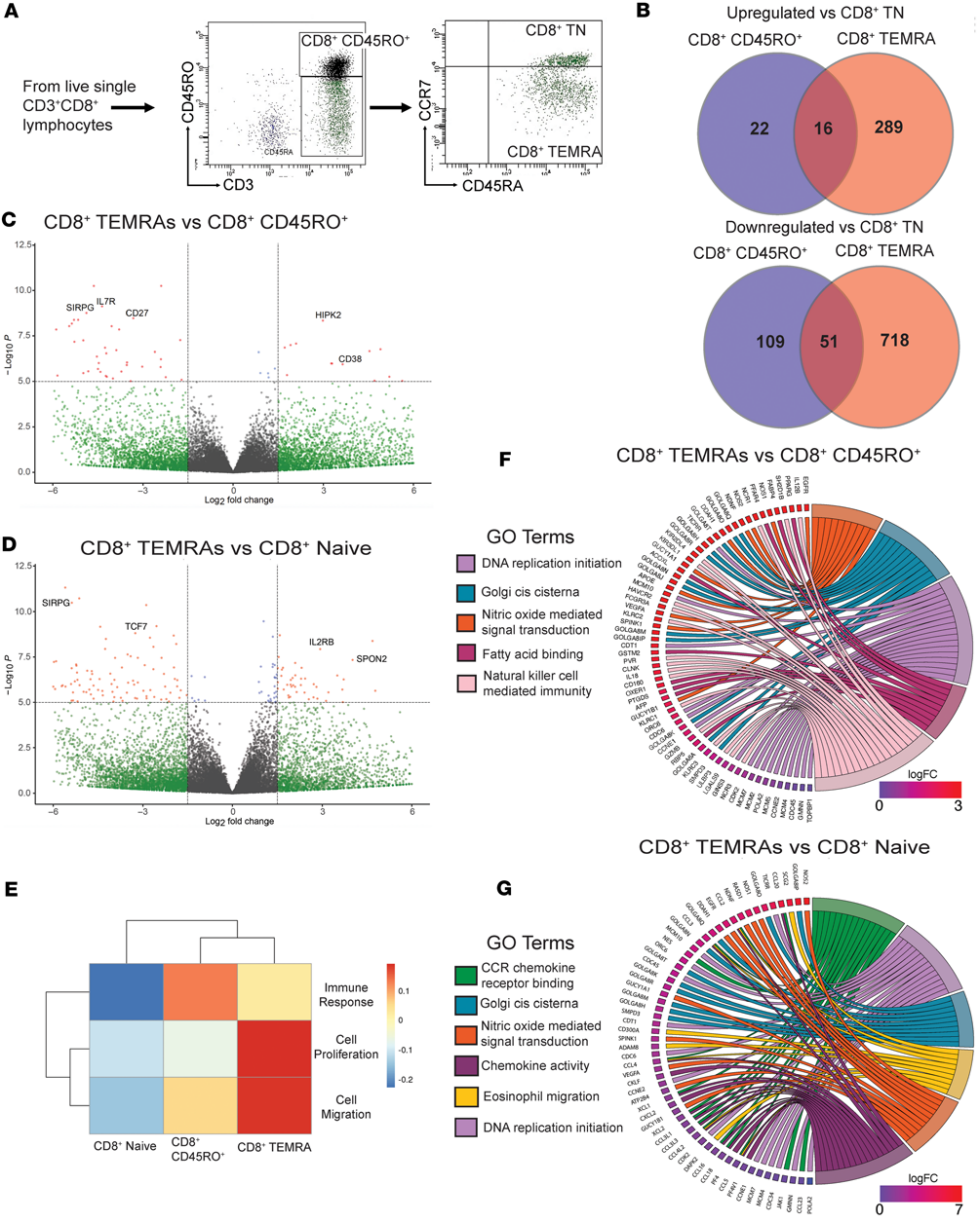
Transcriptional features of PB CD8^+^ TEMRAs in SA suggest inflammatory phenotype and a role for IL-15. (**A**) Representative gating strategy for flow sorting 3 CD8^+^ T cell populations from patients with SA: naive, CD45RO^+^ memory, and TEMRAs. (**B**) Venn diagrams illustrating the number of genes differentially upregulated (left) or downregulated (right) versus CD8^+^ naive T cells. (**C**) Volcano plot illustrating DEGs in sorted CD8^+^ TEMRAs versus CD8^+^ CD45RO^+^ memory. (**D**) Volcano plot illustrating DEGs in sorted CD8^+^ TEMRAs versus CD8^+^ naive T cells. (**E**) Heatmap illustrating median enrichment scores for 3 GO terms using GSVA. (**F**) Chord plot representation showing core genes within enriched pathways corresponding to the GO terms shown for CD8^+^ TEMRA versus CD45RO^+^ memory comparisons revealed by GSEA. (**G**) Chord plot representation showing core genes within enriched pathways corresponding to the GO terms shown for CD8^+^ TEMRA versus CD8^+^ naive T cell comparisons revealed by GSEA.

To further explore CD8^+^ TEMRAs in patients, we performed bulk RNA sequencing (RNA-seq) on sorted PB CD8^+^ TEMRAs, CD8^+^CD45RO^+^ memory, and CD8^+^ naive T cells isolated from 3 patients with SA (Figure 3A). Cells were not stimulated with anti-TCR or phorbol myristate acetate with ionomycin prior to cell sorting. We examined the number of differentially expressed genes (DEGs), both upregulated and downregulated, in SA CD8^+^ TEMRAs compared to SA naive CD8^+^ T cells that were non-overlapping when a similar comparison was made between the transcriptomes of SA CD8^+^CD45RO^+^ T cells and SA naive CD8^+^ T cells. When compared with CD8^+^ naive T cells, CD8^+^ TEMRAs differentially upregulated 289 genes and differentially downregulated 718 genes that were not shared with CD8^+^CD45RO^+^ memory T cells (Figure 3B). These results, combined with earlier biomarker and cytokine experiments, suggested that CD8^+^ TEMRAs are differentially programmed from other canonical memory T cell populations in SA. We analyzed the data further to identify specific DEGs and biological pathways in CD8^+^ TEMRAs compared to the other 2 cell types. As shown in Figure 3C, lower expression of the gene *SIRPG* in CD8^+^ TEMRAs compared with CD8^+^CD45RO^+^ T cells was observed. This may lower the threshold of TEMRA activation in SA given that a single nucleotide polymorphism in this gene was previously associated with increased CD8^+^ effector T cell activity with promotion of autoimmunity (48). CD8^+^ TEMRAs differentially downregulated *IL7R* (CD127) and *CD27* (Figure 3C) that was also evident at the protein level (Figure 2C). Although IL-7 is critical for the maintenance of naive and memory T cells (49, 50), TEMRAs may be less dependent on IL-7 for their maintenance. Instead, differential upregulation of *IL2RB* in CD8^+^ TEMRAs compared with CD8^+^ naive T cells (Figure 3D) suggested that these cells are wired to respond to both IL-2 and IL-15, as IL-2Rβ is a shared signaling subunit for both of these cytokines in the induction of T cell proliferation (51, 52). As such, compared with CD4^+^ T cells, a higher pool of IL-2R in CD8^+^ T cells was reported in a study that also demonstrated greater CD8^+^ T cell proliferation in response to IL-2 (53). Whereas canonical CD8^+^ effector memory T cells sustain their antiviral effects through either IL-2 or IL-15, we found that CD8^+^ TEMRAs differentially upregulated *CD38* compared with CD8^+^CD45RO^+^ T cells (Figure 3C), which may dampen their antiviral activity, increased *CD38* expression having been previously shown to reduce cytotoxic functions of CD8^+^ T cells against viral infection in patients with lupus (54). However, increased expression of *HIPK2*, associated with type I IFN–mediated antiviral responses (55), may offset higher *CD38* expression in the TEMRAs. IL-15 is known to maintain basal proliferation of memory CD8^+^ T cells (56, 57). Downregulation of *TCF7* (which encodes the transcription factor TCF-1), observed in CD8^+^ TEMRAs, was also previously associated with CD57^+^ cells, a feature of cellular senescence (Figure 3D) (58). Finally, when compared with CD8^+^ naive T cells, CD8^+^ TEMRAs also differentially upregulated *SPON2*, which encodes the extracellular matrix protein spondin 2. *SPON2* gene expression is associated with leukocyte migration (59) and its increased expression was observed in CMV-specific CD8^+^ T cells (60).

The profile of DEGs in CD8^+^ TEMRAs suggested differential enrichment of biological processes in these cells compared with the other 2 cell types. Toward this end, we performed gene set variation analysis (GSVA), which is an unsupervised non-parametric algorithm that can score enrichment of a specific pathway across multiple samples. We focused on 3 broad categories — immune response, cell proliferation, and cell migration, which are based on gene sets within these gene ontology (GO) terms. This analysis revealed that the overall enrichment score in CD8^+^ TEMRAs for immune response was higher than in naive T cells but less than that in CD45RO^+^ T cells (Figure 3E). TEMRAs scored higher than both for cell proliferation and cell migration (Figure 3E). Subsequent gene set enrichment analysis (GSEA) revealed that CD8^+^ TEMRAs, whether compared with CD45RO^+^ cells or naive T cells, showed enrichment of nitric oxide (NO) signaling, DNA replication initiation, and *cis*-Golgi cisterna (Figure 3F). Enrichment of the *cis*-Golgi cisterna gene set in the TEMRAs suggested increased secretory propensity in these cells (61). NO signaling was previously reported to promote a Th1 response (62), which we also found in CD8^+^ TEMRAs skewed toward T1 cytokine secretion (Figure 2, A and B). We recently reported high levels of fractional exhaled NO (FeNO) in patients with SA with a high sputum CCL5 signature, CCL5 correlating with both T1 and T2 inflammatory responses (35). In line with the higher IFN-γ signature, we also observed enrichment of the GO term associated with NK cell–mediated immunity in CD8^+^ TEMRAs when compared with CD8^+^CD45RO^+^ cells (Figure 3F). In a previous study, CD8^+^ T cells were observed to upregulate IL-15–induced activating NK receptors in a TCR-independent manner in patients with acute viral infection (63). In addition, IL-15 was reported to induce migration of CD8^+^ TEMRAs into non-lymphoid tissue (41) and enhance CCR5-mediated migration of memory CD8^+^ T cells (64).

When compared with naive CD8^+^ T cells, CD8^+^ TEMRAs displayed enrichment of pathways involved in chemokine-chemokine receptor interaction and eosinophil migration (Figure 3G). Thus, enrichment of these gene sets along with that for NO signaling in the CD8^+^ TEMRAs suggests that these cells are important players in the interaction between T1 and T2 pathways, a feature of the sickest of patients in SA (35, 37). An additional pathway that was enriched in the CD8^+^ TEMRAs was DNA replication initiation, which was associated with a high level of TICRR expression, suggesting that these cells are poised for DNA synthesis and cell proliferation. TICRR is an essential checkpoint and replication regulator (65) and is upregulated in all types of cancer with poor prognosis (66). Enrichment of the fatty acid binding gene set in TEMRAs compared with CD45RO^+^ cells suggested that like TRMs, TEMRAs may also rely on fatty acid metabolism for survival (67).

Collectively, these results showed that CD8^+^ TEMRAs harbor a transcriptional profile that favors cell migration, proliferation, secretion, and increased NK-like effector function, including IFN-γ production. In addition, the cells may promote interaction between T1 and T2 immune responses in SA. While the expression of cell surface markers in TEMRAs shown in Figure 2 suggested that TEMRAs are senescent and thus likely unable to proliferate, analysis of their transcriptome suggested otherwise. Recent studies have also questioned complete cell cycle arrest in CD8^+^ TEMRAs (42, 68, 69). In aggregate, the data suggested that CD8^+^ TEMRAs have phenotypic overlap with NK cells and may be sensitive to IL-15 stimulation. Given that IL-15 can recruit TEMRAs into non-lymphoid tissue (41), induce TEMRA proliferation (42), and is associated with asthma exacerbations (70), we examined *IL15* expression in airway brushings from patients with MMA and SA.

### IL15 is upregulated in patients with SA compared with MMA and correlates with T cell surface markers.

IL-15 is a chemokine and activator of short-lived effector CD8^+^ T cells, CD8^+^ effector memory T cells, and CD8^+^ TEMRAs (57, 71). IL-15 signals primarily via *trans* presentation after complexing with the by the α subunit of the IL-15 receptor (IL-15Rα) and has been postulated to be a T1 alarmin cytokine, along with type 1 IFNs, which can serve to costimulate CD8^+^ effectors in peripheral tissues (72). In addition, antigen-independent expression of IL-15 and IL-15–regulated genes was overrepresented in an Ingenuity Pathway Analysis of PBMCs isolated from patients with asthma exacerbations, suggesting this cytokine may play a role in antigen-independent cell stimulation (70). In the lung, potential sources of IL-15 include damaged epithelial cells, monocytes, macrophages, and dendritic cells. We assessed expression of *IL15* and the gene for the IL-15R α subunit (*IL15RA*) in the airways of SA and MMA patients.

**Figure 4:**
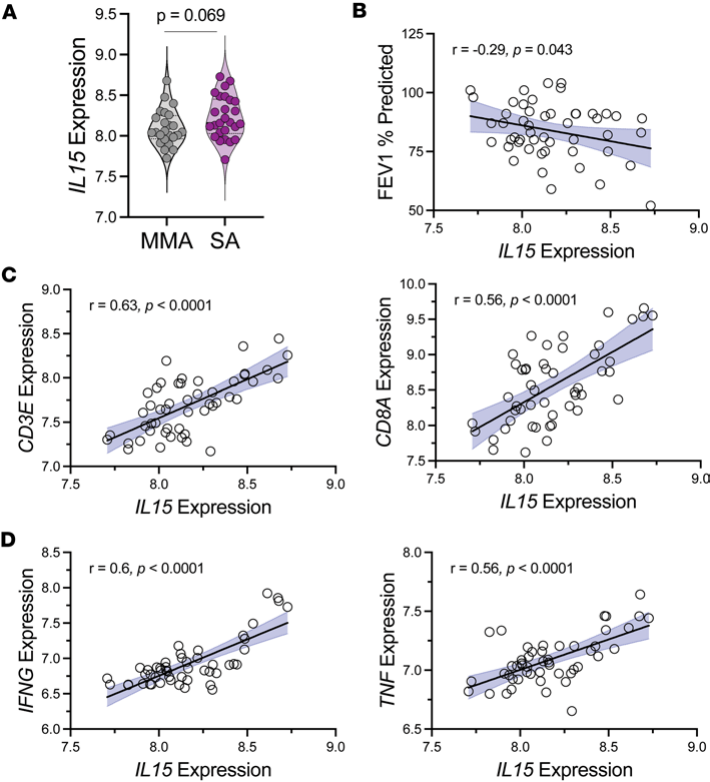
*IL15* expression in bronchial brushings implicates IL-15 in disease pathogenesis in SA. (**A**) *IL15* gene expression in cells isolated from endobronchial brushings. (**B**) Linear regression of percentage predicted forced expiratory volume in 1 second (FEV1) as a function *IL15* gene expression. (**C**) Linear regression of *CD3E* gene expression (left) or *CD8A* gene expression (right) as a function *IL15* gene expression. (**D**) Linear regression of *IFNG* gene expression (left) or *TNF* (right) as a function *IL15* gene expression. Statistical significance for data shown in **A** determined using Mann-Whitney test and for panels **B**–**D** using Spearman’s nonparametric correlations, with solid black lines representing simple linear regression line and shaded blue area representing 95% confidence interval.

Targeted bioinformatic analysis of previously generated RNA-seq data of bronchial brushings from patients in the Immune Mechanisms of Severe Asthma (IMSA) cohort was performed as previously described (73). These participants also had paired clinical metadata available, including pulmonary function testing. We detected a trend toward higher *IL15* expression (*P* = 0.069) in patients with SA compared with MMA (Figure 4A). Interestingly, *IL15* expression negatively correlated with the percentage predicted FEV1, a measure of airway obstruction in asthma (Figure 4B). There was a significant positive correlation between *IL15* expression and markers of T cells, including *CD3E* and *CD8A* (Figure 4C) but did not include *CD4* (Supplemental Figure 2E), suggesting the presence of nested CD8^+^ T cells in the airways of patients with asthma as previously observed (74, 75). *IL15* expression also positively correlated with *IL15RA* expression (Supplemental Figure 2F) in patients with asthma, which could represent *trans* presentation of IL-15 by damaged airway cells. IL-15 is not only a chemokine and activator of effector and effector memory T cells, but it can also skew these cells toward a T1 phenotype (41, 42). Indeed, we found a significant positive correlation between expression of *IL15* and *IFNG* as well as *TNF* (Figure 4D). Taken together, these data suggested that higher IL-15 expression in patients with SA, but not MMA, may serve as a chemokine and activator of CD8^+^ TEMRAs, which may lead to more local immunopathology and lower lung function.

### IL-15R is detectable in the airways of patients with SA.

**Figure 5:**
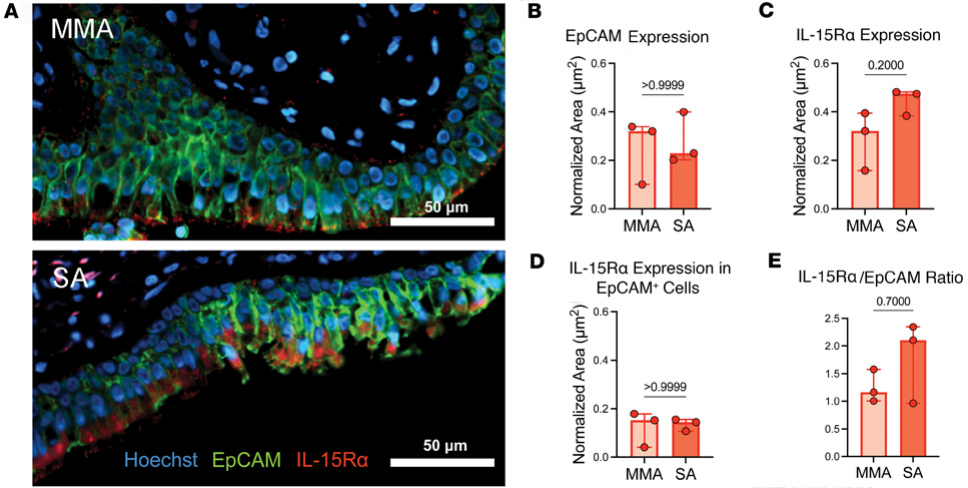
IL-15Rα is detectable by immunofluorescence in the airways of patients with SA. (**A**) Immunofluorescence microscopy of endobronchial biopsy specimens from patients with mild to moderate asthma (top) and severe asthma (bottom). Images are representative of *n* = 3 from each group. Tissues were stained for IL-15Rα (red), EpCAM (green), and were counterstained with Hoechst (blue) to highlight nuclei. Scale bars: 50 μm. Plots show (**B**) normalized area of EpCAM, (**C**) IL-15Rα, (**D**) IL-15Rα expression in EpCAM^+^ cells, and (**E**) the ratio of IL-15Rα to EpCAM expression. Quantification of immunofluorescence labeling corrected for background signal. Statistical significance for data in panels **B**–**E** determined using Mann-Whitney test. Data in panels **B**–**E** represent median ± 95% confidence interval.

To validate the findings of increased *IL15* expression and an association with lower airway function in patients with SA, we investigated whether IL-15Rα protein expression was detectable in endobronchial biopsy specimens obtained from donors in the IMSA cohort. We investigated IL-15Rα, rather than the IL-15 protein, as *IL15RA* correlated with *IL15* in our transcriptional data and, as an extracellular protein, IL-15Rα quantification is technically easier using our endobronchial biopsy specimens. Formalin-fixed and paraffin-embedded endobronchial biopsy specimens were obtained from patients with MMA (*n* = 3) and SA (*n* = 3) and stained with antibodies against an epithelial cell marker (EpCAM) and IL-15Rα. We found that IL-15Rα expression was detectable in both patients with MMA and SA (Figure 5A). When normalized area of protein expression was calculated, there was no difference in normalized EpCAM expression between MMA and SA (Figure 5B) yet there was a trend toward increased expression of IL-15Rα in SA (Figure 5C). Although with our limited sample size, there was no statistically significant difference between normalized area of IL-15Rα expression among EpCAM^+^ cells (Figure 5D) or the IL-15Rα/EpCAM ratio (Figure 5E) in the airways of patients with MMA or SA, the data suggest that epithelial cells may not be the only airway source of IL-15Rα. Regardless, these results confirm that IL-15Rα is present in the airways of patients with SA and therefore could influence CD8^+^ TEMRA biology by *trans* presentation of IL-15. Most importantly, even if IL-15 can be potentially presented in *trans* in the airways of MMA patients, IL-15 does not enhance TCR-induced proliferation of CD8^+^ TEMRAs obtained from these patients, unlike what we observe for the corresponding cells from the patients with SA. The mechanism(s) underlying this interesting dichotomy remains to be determined in future studies.

### PB CD8^+^ TEMRAs retain proliferative capacity in patients with SA but not MMA.

To determine bona fide senescence leading to irreversible cell-cycle arrest (20), we cultured PBMCs, stimulated them with a cocktail of bead-conjugated antibodies against CD2, CD3, and CD28 at a low (TCR^lo^) or high (TCR^hi^) bead to cell ratio for 5 days, and then evaluated for proliferation of different T cell subsets by flow cytometry. Cells were either stimulated via TCR alone, or in combination with IL-2, IL-15, or both IL-2 and IL-15. Memory T cells have been shown to respond to IL-15 stimulation through *trans* presentation of IL-15 complexed to the IL-15R α subunit, a common mechanism of IL-15 action on target cells (71). We used soluble IL-15 as a stimulus condition, as studies have shown that human memory T cells, including TEMRAs, do not require *trans* presentation of IL-15 in vitro (42, 76).

**Figure 6:**
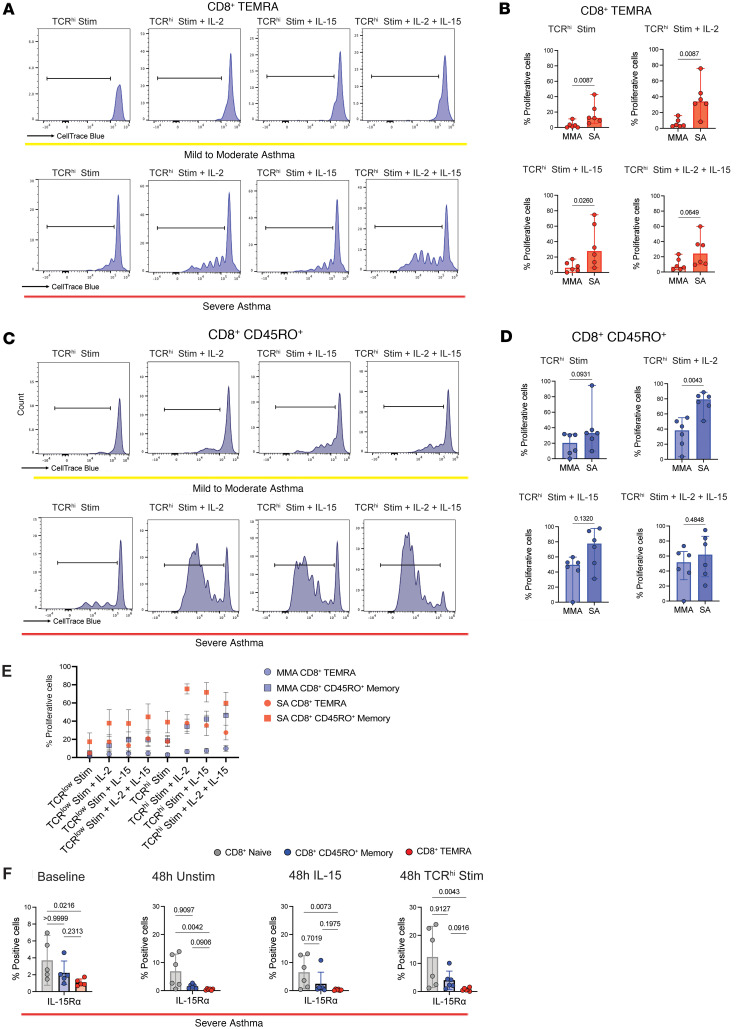
PB CD8^+^ TEMRAs retain proliferative capacity in SA, but not MMA, despite senescent phenotype of both. (**A**) Histograms of CellTrace Blue dye in CD8^+^ TEMRAs isolated from patients with MMA (top) or SA (bottom). (**B**) Percentage of PB CD8^+^ TEMRAs proliferating as measured by CellTrace Blue dye under 4 stimulation conditions. (**C**) Histograms of CellTrace Blue dye in CD8^+^CD45RO^+^ memory T cells isolated from patients with MMA (top) or SA (bottom). (**D**) Percentage of PB CD8^+^CD45RO^+^ memory T cells proliferating as measured by CellTrace Blue dye under 4 stimulation conditions. (**E**) Dot plot showing T cell proliferation responses to different stimulation conditions in patients with MMA (blue points) or SA (red points). Each point represents the mean value (*n* = 6), with error bars representing the standard error of the mean. (**F**) Percentage of IL-15Rα–expressing PB cells from patients with SA at baseline, 48 hours in culture, 48 hours in culture with IL-15, or 48 hours in culture with TCR^hi^ stimulation. Statistical significance for data shown in **B** and **D** determined using Mann-Whitney test and for data shown in **F** determined using Kruskal-Wallis followed by multiple pairwise comparisons. Data in **B**, **D**, and **F** represent median ± 95% confidence interval.

While CD8^+^ TEMRAs in patients with MMA exhibited a senescent phenotype with few proliferative cells across all conditions, TEMRAs in patients with SA proliferated at significantly higher rates (Figure 6A). SA CD8^+^ TEMRAs ranged between a mean of 17.6% proliferative cells in the TCR^hi^ stimulation condition to 38% proliferative cells in the TCR^hi^ stimulation plus IL-2 (Figure 6B). SA CD8^+^ TEMRAs retained proliferative capacity despite expression of senescence biomarkers, which suggested these cells were not truly senescent (68). It is now well recognized that a cell cannot be deemed as senescent solely based on expression of senescence-associated molecules (20). We also assayed the proliferative potential of CD8^+^CD45RO^+^ T cells, which were much more proliferative across all conditions when compared with CD8^+^ TEMRAs (Figure 6C). CD8^+^CD45RO^+^ T cells isolated from patients with SA also proliferated at a higher rate when compared with those from patients with MMA, which may reflect a lower threshold for proliferation for all memory T cells in SA (Figure 6D). It is worth noting that despite the higher proliferative potential of CD8^+^CD45RO^+^ T cells compared with CD8^+^ TEMRAs, only the latter in the patients with SA showed a positive correlation with asthma duration, especially in the case of the BAL cells (Figure 1, D and E). It is possible that the local microenvironment in the airways of SA patients drives a proportional increase in CD8^+^ TEMRAs at the expense of CD45RO^+^ T cells over time, TEMRAs being terminally differentiated memory T cells (23, 24), thus explaining their selective association with asthma duration. In TCR^lo^ conditions, the addition of both IL-2 and IL-15 was sufficient to increase SA CD8^+^ TEMRA proliferation compared with TCR^lo^ stimulation alone, suggesting that the presence of IL-15 or IL-2 in vivo may augment proliferation of these inflammatory cells (Figure 6E). IL-2 or IL-15 may act as cofactors for TCR stimulation in peripheral tissues, which has been a hypothesized activation mechanism (72).

Finally, we followed these proliferation experiments with an assessment of IL-15R expression on PB T cell subsets at unstimulated baseline and under various conditions at 48 hours in culture. Consistent with prior literature, at baseline, a significantly smaller percentage of SA CD8^+^ TEMRAs expressed IL-15R (Figure 6F). Unstimulated T cells left in culture for prolonged periods of time undergo apoptosis and therefore we were only able to measure IL-15R in vitro at 48 hours. PBMCs were split between unstimulated conditions or stimulation with TCR^hi^ stimulation alone or IL-15 alone. We found that across all conditions, SA CD8^+^ TEMRAs in PB express low levels of IL-15R. Relevant to our observations, a prior study showed internalization of cell surface–bound IL-15/IL-15R complex in CD8^+^ T cells and LPS- or IFN-γ–activated monocytes, with recycling of the complex to the cell surface (77). This mechanism was proposed to support long-term survival of CD8^+^ memory T cells in the absence of IL-15 and also allow *trans* presentation of IL-15 by monocytes to T cells expressing only the β subunit of IL-2R (IL-2Rβ) and the common γ chain (γc) shared by multiple cytokine receptors, including IL-2 and IL-15. Thus, it is possible that a combination of mechanisms facilitated the proliferative response to soluble IL-15 in our experiments, including baseline expression of IL-15R in a small percentage of the CD45RO^+^ memory and TEMRAs and *trans* presentation of IL-15 to T cells by monocytes present in the PBMC cultures.

## Discussion

Although Th2 cells are most commonly associated with asthma pathogenesis, in recent years, we and others have described a heightened T1/IFN-γ^+^ immune response, often mixed with T2 cells, in the airways of patients with SA (6, 10, 12, 33, 34, 37). These studies revealed different memory T cell subsets, including TRMs, to be an important source of IFN-γ in the airways of a subset of severe asthma patients (6, 12). However, IFN-γ^+^ TRMs were also detected in a subset of patients with mild to moderate disease (6). Thus, the contribution(s) of T cell types to severe versus milder disease has remained unclear. We therefore investigated how different memory T cells associate with the onset of disease symptoms in severe versus milder asthma, which had not been examined heretofore to our knowledge. This question also addressed a key feature of asthma, which is its chronicity. We focused on the terminally differentiated memory T cell, TEMRA, due to its association with multiple chronic inflammatory diseases in recent years. Our study has revealed that IFN-γ^+^CD8^+^ TEMRAs in the PB and BAL of patients with SA, but not MMA, correlate with asthma duration since the onset of disease symptoms. A second feature that functionally distinguished CD8^+^ TEMRAs in severe versus milder disease was the ability of cells from patients with SA to proliferate ex vivo unlike those from patients with MMA, although the cells from both patient groups displayed similar profiles of expression of cellular-senescence-associated cell surface markers. While IL-15, a cytokine known to maintain and promote proliferation of CD8^+^ T cells, further enhanced TCR-induced proliferation of TEMRAs from patients with SA, it failed to release the proliferation block in the TEMRAs of patients with MMA. The trend toward higher *IL15* expression in the airways of patients with SA compared with MMA, airway expression of IL-15Rα, and the inverse relationship of *IL15* expression with lung function in the patients with asthma focuses attention on an IL-15/CD8 TEMRA axis in asthma disease severity and chronicity not yet appreciated in the existing literature.

We did not detect an increase in the frequency of BAL TRM cells within the CD8^+^ T cell pool over the same timeframe. This raises fundamental questions about mechanisms that selectively promote and maintain a pool of IFN-γ^+^CD8^+^ TEMRAs with enhanced cytotoxic functions in the airways of patients with more severe disease. In light of our present and previous findings, it would be particularly important to determine whether CD8^+^ TRMs and CD8^+^ TEMRAs are functionally distinct with regard to expression of markers of senescence and the SASP phenotype. SASP protects from tumorigenesis but chronic cytokine secretion in a tissue can result in age-associated chronic inflammation or inflammaging (15, 22). However, SASP can occur in non-senescent cells as well (20). One feature of senescent cells is increased p38 MAP kinase activity (68). It is possible that TEMRA-associated p38 MAP kinase activity is different in severe compared with mild to moderate disease and may provide an explanation for the observation that BAL CD8^+^ TEMRAs associate with disease chronicity only in patients with SA. It is also possible that there is more IL-15–mediated homeostatic proliferation of TEMRAs in the airways of patients with SA compared with MMA, as IL-15 is known to promote basal proliferation of memory CD8^+^ T cells (56, 57).

CD8^+^ TEMRAs are increasingly being associated with a number of diseases, including allograft rejection (42), autoimmune disease (25, 78), and, as our current study shows, severe asthma. The virus most frequently associated with TEMRA generation is cytomegalovirus (CMV) (27, 28). Recently, increased CMV reactivation was associated with high CS use in patients with severe acute respiratory syndrome coronavirus 2 (SARS-CoV-2) (79) and is a known concern among immunosuppressed organ transplant recipients (80). It is possible that inhaled or oral CS use in some patients with SA may induce reactivation of CMV (or another virus) in the airways of these patients. Notably, increased viral carriage has been observed in the airways of patients with SA (37). Over time, persistent stimulation by viral antigens may drive TEMRA accumulation and IL-15–mediated activation.

IL-15 has many known effects on CD8^+^ T cells, including stimulation of basal proliferation, chemoattraction, and promotion of bystander activation. It is responsible for the coordination of a protective immune response during defense against viral pathogens (81). However, in the present study, we demonstrate a potentially detrimental effect of high *IL15* expression in the airways of SA patients with its inverse association with lung function, as measured by FEV1. This invites the hypothesis that dysregulated expression of IL-15 may be a mediator of disease pathogenesis in SA and, more insidiously, portend the development of T1-associated diseases following the diagnosis of asthma (82). IL-15 was one of the few cytokines significantly associated with autoantibodies isolated from the sputum of individuals with severe asthma (83) and IL-15–mediated CD8^+^ memory T cell development is being increasingly recognized in various disease settings (84). Tissue damage caused by increased numbers of CD8^+^ T cells has been attributed to increased NK-like activity (63), which we also detected in the TEMRA population in our GSEA (Figure 5F). Future studies will need to assess whether the senescence-associated features in the CD8^+^ TEMRAs in a subset of SA patients induce SASP causing premature airway aging (21). Given the ability of IL-15 to stimulate bystander activation of CD8^+^ T cells, targeting this cytokine is being considered in organ transplant recipients and in those with autoimmune disease (85). Almost all biologics currently available in the treatment of SA are targeted against molecules associated with the T2 pathway (86), yet an expanding body of literature shows that immune dysfunction in SA is more complex than just involving an aberrant T2 immune response. As we previously reported, high levels of CCL5 in the airways of patients with a mixed T1-T2 immune response (35), highlights the CCL5 receptor, CCR5, as one such attractive target. Importantly, CCL5 is a component of multiple pathways enriched in the CD8^+^ TEMRAs, including eosinophil migration (Figure 3G), suggesting that TEMRAs in conjunction with the CCL5/CCR5 axis may serve to bridge T1-T2 communication in SA.

In summary, we found a strong association between asthma duration and CD8^+^ TEMRAs in the airways of patients with SA. Compared with that in patients with MMA, higher percentages of IFN-γ^+^CD8^+^ TEMRAs were detected in the PB and BAL of patients with SA. However, despite showing similar profiles of expression of senescence-associated biomarkers, the TEMRAs from patients with SA, but not MMA, responded to proliferative cues ex vivo. Our data suggest an intimate relationship between TEMRAs and IL-15 expression in the airways of patients with SA, showing a trend toward higher expression compared with patients with MMA. The positive associations of IL-15 with the maintenance and abundance of CD8^+^ memory T cells and with asthma exacerbations coupled with the negative relationship with lung function, as revealed in the present study, prompt us to propose future investigations of IL-15 as a potential therapeutic target in SA.

## Methods

### Sex as a biological variable.

Sex was considered as a biological variable when these experiments were designed. However, due to the biological heterogeneity in patients with asthma and the difficulty in obtaining specimens from well-characterized donors, we found that we were underpowered to distinguish the effect of sex on observed phenotypes in this study.

### Human participants in the IMSA cohort.

Briefly, non-smoking subjects were included in the IMSA study if they were at least 18 years of age. Patients with MMA or SA were recruited if they had a confirmed diagnosis of asthma. Diagnosis of asthma was established in accordance with international guidelines (87) and with clinical characteristics combined with either a positive bronchodilator response or a positive methacholine challenge. Study participants underwent extensive characterization including donation of peripheral blood, pulmonary function testing, and bronchoscopy with collection of BAL fluid. Bronchoscopy was performed per IMSA published protocols and consistent with procedures established at the University of Pittsburgh and by the Severe Asthma Research Program (SARP). Cells were cryopreserved and samples were assayed in bulk. Demographic information about the participants in Figure 1 is included in Supplemental Table 1. Demographic data about participants from whom additional samples were used (Figures 2–6) are not included in Supplemental Table 1 and are available upon request.

### Flow cytometric immunophenotyping.

Cryopreserved cell suspensions were thawed, resuspended in media, and then stimulated with phorbol myristate acetate and ionomycin (both Sigma-Aldrich) at 37°C for 2.5 hours in the presence of monensin and brefeldin A (both BioLegend). Cells were washed, resuspended in FACS buffer, and incubated with optimally diluted labeled mAbs for 30 minutes at 0°C. Cells were washed, fixed overnight, and then resuspended in permeabilization buffer. Staining for intracellular proteins using optimally diluted mAbs was performed for 1 hour at 0°C. Cells were washed, fixed, resuspended in FACS buffer, and data acquisition was performed on an Aurora flow cytometer (Cytek).

### Mass cytometry (CyTOF).

Mass cytometry data were reanalyzed from a dataset created as previously described (6) and are publicly available in the FlowRepository at https://flowrepository.org/id/FR-FCM-Z395 Briefly, BAL cells were thawed, resuspended in media, and stimulated prior to incubation with mAbs conjugated to rare earth heavy metal isotopes for 45 minutes at 0°C. Cells were washed, fixed overnight, resuspended in permeabilization buffer, and incubated with metal-labeled mAbs directed against intracellular proteins as described above. Single-cell mass spectrometry data were acquired using a Helios CyTOF system (Fluidigm). Data analysis for both flow and mass cytometry was carried out using FlowJo software (Tree Star).

### FACS.

Cryopreserved cell suspensions were thawed as above and resuspended in FACS buffer, but instead of stimulation were incubated with optimally diluted labeled mAbs for 30 minutes at 0°C. Cells were washed and then sorted on an Aurora CS cell sorter using SpectroFlo software. Up to 100,000 T cells from 3 populations (CD8^+^ naive, CD8^+^CD45RO^+^ memory, and CD8^+^ TEMRAs) were sorted directly into 1 mL of RLT buffer (Qiagen).

### Bulk RNA-seq library generation and sequencing.

Libraries were generated with the Takara SMART-Seq Stranded kit using Takara SMARTer RNA Unique Dual Index A and B Kits according to the manufacturer’s instructions. RNA was normalized to 5 ng/μL with water for a total volume of 7 μL of input RNA. RNA fragmentation was carried out for 6 minutes. Ten cycles were used for PCR1, followed by ribosomal RNA depletion using scZapR. No samples were pooled prior to PCR2 where 12 cycles were completed. Library quantification and assessment was done using a Qubit FLEX fluorometer and an Agilent TapeStation 4150/Fragment Analyzer 5300. Libraries were normalized and pooled to 2 nM by calculating the concentration based on the fragment size (bp) and the concentration (ng/μL) of the libraries. Sequencing was performed on an Illumina NextSeq 2000, using a P3 200 flow cell. The pooled library was loaded at 750 pM. Sequencing was carried out 2 × 101 bp, with a target of 40 million reads per sample. A custom run chemistry was used to incorporate 3 dark cycles at the start of R2 that mask the 3 bp of the Takara adapter present in the read. Sequencing data was demultiplexed by the on-board Illumina DRAGEN FASTQ Generation software (v3.10.12).

### Bulk RNA-seq data analysis of sorted T cells.

The quality of the raw RNA-seq data was assessed using FastQC (https://www.bioinformatics.babraham.ac.uk/projects/fastqc/), poor quality ends were trimmed using Trimgalore (https://www.bioinformatics.babraham.ac.uk/projects/trim_galore/). High-quality sequences were aligned against the reference genome (hg38) using STAR aligner v2.7.9 (88), and counts were generated using featureCounts (89). B cell–related genes listed in Supplemental Table 2 were removed from the counts data. A list of DEGs was generated between the different conditions using DESeq2 (90) based on the negative binomial distribution. The resulting DEGs between the groups were defined at cutoff criteria of |log_2_ fold-change| of 1.5 or greater and *P* value of less than 0.05 adjusted using the Benjamini and Hochberg approach for controlling the false discovery rate. To extract further biological insight from the samples, we implemented GSEA, which assesses the statistical enrichment of gene ontologies, and pathways using Clusterprofiler v4.8.1 (https://guangchuangyu.github.io/software/clusterProfiler/). Chord diagrams were generated using custom scripts to visualize the GSEA results. All statistical analyses were performed using R 4.2.0 (https://www.r-project.org/). Bulk gene expression data from sorted CD8^+^CD45RO^+^ memory and CD8^+^ TEMRAs were then subjected to GSVA using the GSVA package v1.53.3 (https://github.com/rcastelo/GSVA). GO terms (GO:0008152, GO:0008284, GO:0006955, and GO:0016477) were used for the GSVA. A GSVA score closer to 1 represents upregulation of the pathway, whereas a score closer to –1 represents downregulation of the pathway.

### Transcriptional profiling of endobronchial brushings.

Cells collected from endobronchial brushings during bronchoscopy from patients in the IMSA cohort were collected and subjected to bulk RNA-seq as previously described (91). In brief, RNA isolated from brushings was used to generate cDNA libraries using the Illumina TruSeq RNA Library Prep Kit and cDNA libraries were sequenced on a NextSeq 500 (Illumina). Sequencing data were analyzed using a previously established bioinformatic pipeline (91). Briefly, reads were trimmed using Trimgalore and aligned to Ensemble hg38 using the STAR tool. Gene counts were generated using the featureCounts package and counts were filtered to remove genes with low expression. The DESeq2 package was used to transform the data using a variance stabilizing transformation and then was batch corrected with ComBat (92).

### Proliferation assay.

Cryopreserved cell suspensions were thawed, resuspended in media, and then resuspended in media containing CellTrace Blue dye (Thermo Fisher Scientific) per manufacturer’s protocol for 20 minutes at room temperature. Cells were washed, incubated for an additional 10 minutes at room temperature per manufacturer’s protocol, and then stimulated with either a TCR^lo^ (1 bead per 20 cells) or TCR^hi^ (1 bead per 2 cells) bead stimulation cocktail including anti-CD2, anti-CD3, and anti-CD28 beads (Miltenyi Biotec). Cell conditions included stimulation alone, stimulation with the addition of IL-2 (PeproTech) at 20 g/mL concentration, stimulation with the addition of IL-15 (PeproTech) at 20 g/mL concentration, or stimulation with both IL-2 and IL-15. Cells were stimulated in a 24-well plate in 2 mL X-Vivo 15 Serum Free media (Lonza Bioscience) for 5 days in an incubator at 37°C with 5% CO_2_. After 48 hours in culture, the media were refreshed and IL-2 at a 20 g/mL concentration was added a second time. After 5 days in culture, cells were washed, fixed, resuspended in FACS buffer, stained with a high-dimensional antibody panel as described above (Supplemental Table 3), and data acquisition was performed on an Aurora flow cytometer (Cytek).

### Tissue preparation of endobronchial biopsy specimens and immunofluorescent staining.

Endobronchial biopsies were obtained during bronchoscopy from patients in the IMSA cohort during research biopsies. Formalin-fixed and paraffin-embedded biopsy specimens were cut at 5 μm thickness using a rotary microtome and the sections were mounted onto glass slides. The slides were heated overnight on a heating block at 60°C and then rehydrated with xylene followed by decreasing concentrations of ethanol. Antigen retrieval was performed in citrate buffer at 90°C prior to blocking. Primary antibodies used for immunofluorescence are listed in Supplemental Table 2. To prevent autofluorescence, slides were illuminated with an LED lamp during blocking at room temperature for 2 hours. After rinsing with PBS, tissues were incubated with TrueBlack (Cell Signaling Technology) in 70% ethanol for 1 minute followed by PBS washes. Tissues were then incubated with primary antibodies in a blocking buffer solution overnight at 4°C followed by additional washing steps. Tissues were then incubated with secondary antibodies in blocking buffer for 90 minutes at room temperature. Finally, the tissues were incubated with Hoechst (Thermo Fisher Scientific) for 5 minutes at room temperature, were washed with PBS, and were mounted with Hydromount (National Diagnostics). Primary and secondary antibodies used are listed in Supplemental Table 2.

### Immunofluorescence image acquisition and analysis.

Immunofluorescence images were obtained on a Zellscanner ONE (Canopy Biosciences). Images were then analyzed using the GA3 toolset in NIS Elements (Nikon Inc.). Each channel (Hoechst, Alexa Fluor 488, and phycoerythrin [PE]) was segmented based on intensity, cell size, and shape to generate binary masks identifying positive signal. Using the binary masks, cells positive for either EpCAM or IL-15R were identified using a Boolean “having” statement (i.e., area positive for blue “and” green, or red). EpCAM^+^ cells expressing IL-15R were identified using a Boolean “and” statement (i.e., EpCAM positive cell “and” IL-15R area). Total object count and/or area for each binary mask (nuclei, EpCAM^+^ cells, IL-15R^+^ cells, and EpCAM^+^ cells containing IL-15R) were calculated and final data were normalized to total cell count (Hoechst^+^ objects) to account for differences in total tissue area.

### Statistics.

Statistical analysis of bulk RNA-seq data was performed in R 4.2.0. Otherwise, statistical analysis was performed using Prism v10.2.3 (GraphPad Software). Specific statistical testing procedures, levels of significance, and summary statistics for each experiment are noted in the figures and corresponding legends. A *P* value of less than 0.05 was considered significant.

### Study approval.

All research was approved by the University of Pittsburgh Institutional Review Board (IRB STUDY19030100) and was performed in accordance with all relevant guidelines and regulations. Written informed consent was obtained from all participants prior to inclusion in the IMSA study.

### Data availability.

All cell immunophenotyping and sequencing data presented here are publicly available in alignment with requirements for public disclosure prior to peer review and publication. Mass cytometry data (Figure 1) are publicly available in the FlowRepository at https://flowrepository.org/id/FR-FCM-Z395 Flow cytometry data (Figures 1, 2, and 6) are publicly available in ImmPort under study accession number SDY2766. Bulk RNA-seq data from sorted T cell subsets (Figure 3) will be available through the NCBI Gene Expression Omnibus (GEO GSE273201). Bulk RNA-seq data from endobronchial brushings are available through the NCBI (GEO GSE158752). Raw data are available in the Supporting Data Values file.

## Author contributions

RPR and AR conceived the experimental design, analyzed and interpreted the data, designed the visual representation of data and figures, and wrote the manuscript. RPR, TBO, and JCMP designed the flow cytometry panels, ran flow cytometric immunophenotyping, and sorted T cell subsets. JCMP cultured and stimulated cells in the proliferation experiments. RPR, TBO, SLK, and JCMP analyzed mass and flow cytometry data. NRB, SLK, and MAR performed or advised the imaging work with the endobronchial biopsies and CMSC performed analysis of the imaging data. TJN helped in study participant enrollment, collection of biospecimens, and entry of patient metadata into our database. RPR and SEW analyzed RNA-seq data from endobronchial brushings. SLK, MCG, SD, PR, AVJ, and SEW provided input into experimental design, statistical analysis, figure design, and data interpretation. MY, WC, and DR assisted with the analysis and visualization of bulk RNA-seq data.

## Supplementary Material

Supplemental data

Supporting data values

## Version 1 03/06/2025

In-Press Preview

## Version 2 04/22/2025

Electronic publication
