# Supplementary figures and images for: CD8+ TEMRAs in severe asthma associate with asthma symptom duration and escape proliferation arrest
Source: JCI Insight. 2025 Mar 6;10(8):e185061. doi: 10.1172/jci.insight.185061 (PMC12016929; doi:10.1172/jci.insight.185061)

Supplementary Figure 1

BAL

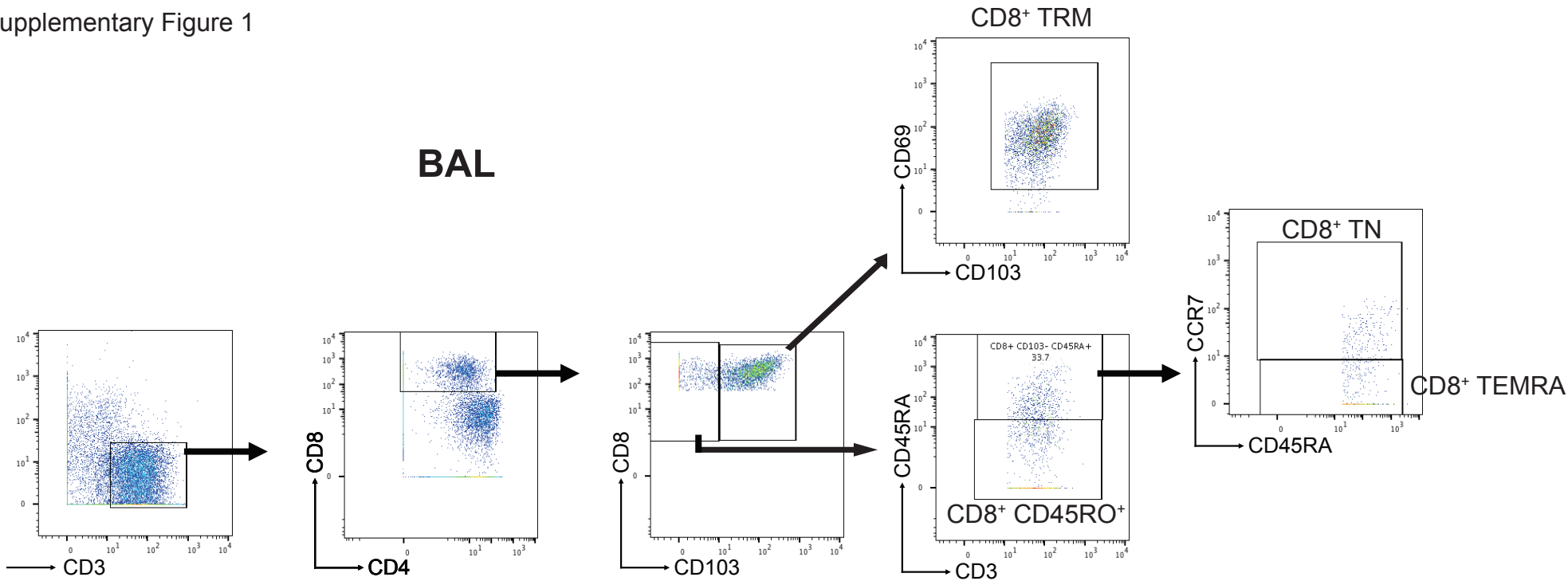

PBMC

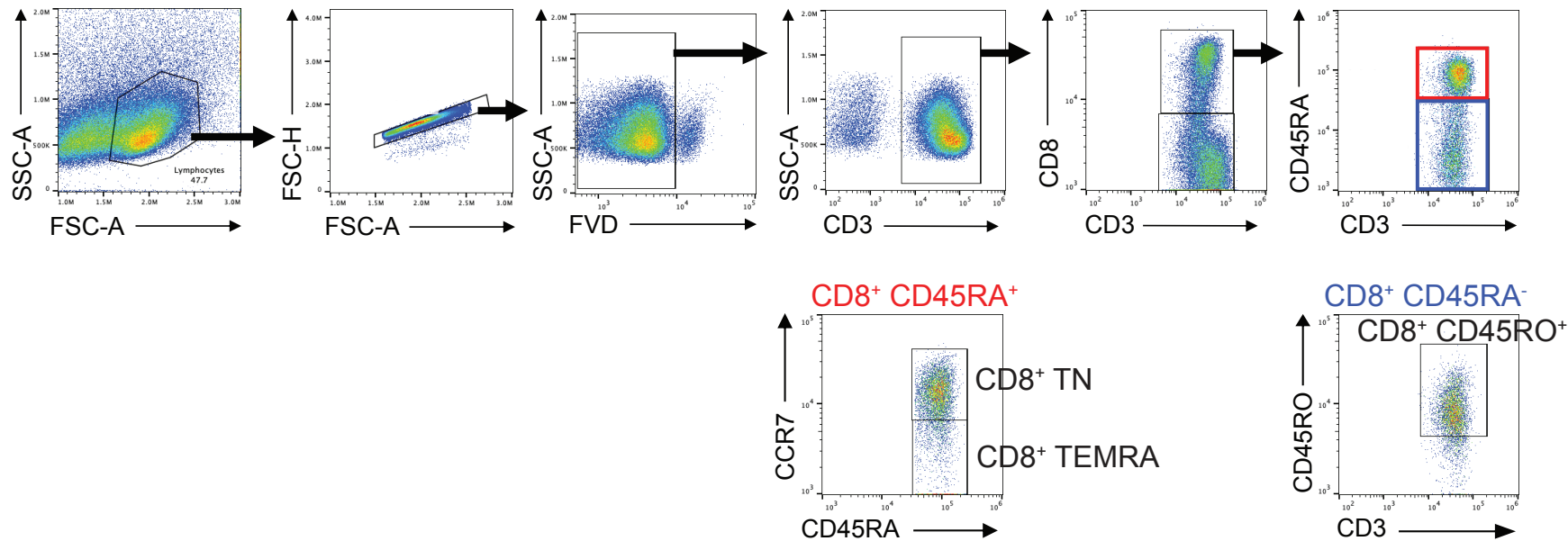

Supplementary Figure 2

A

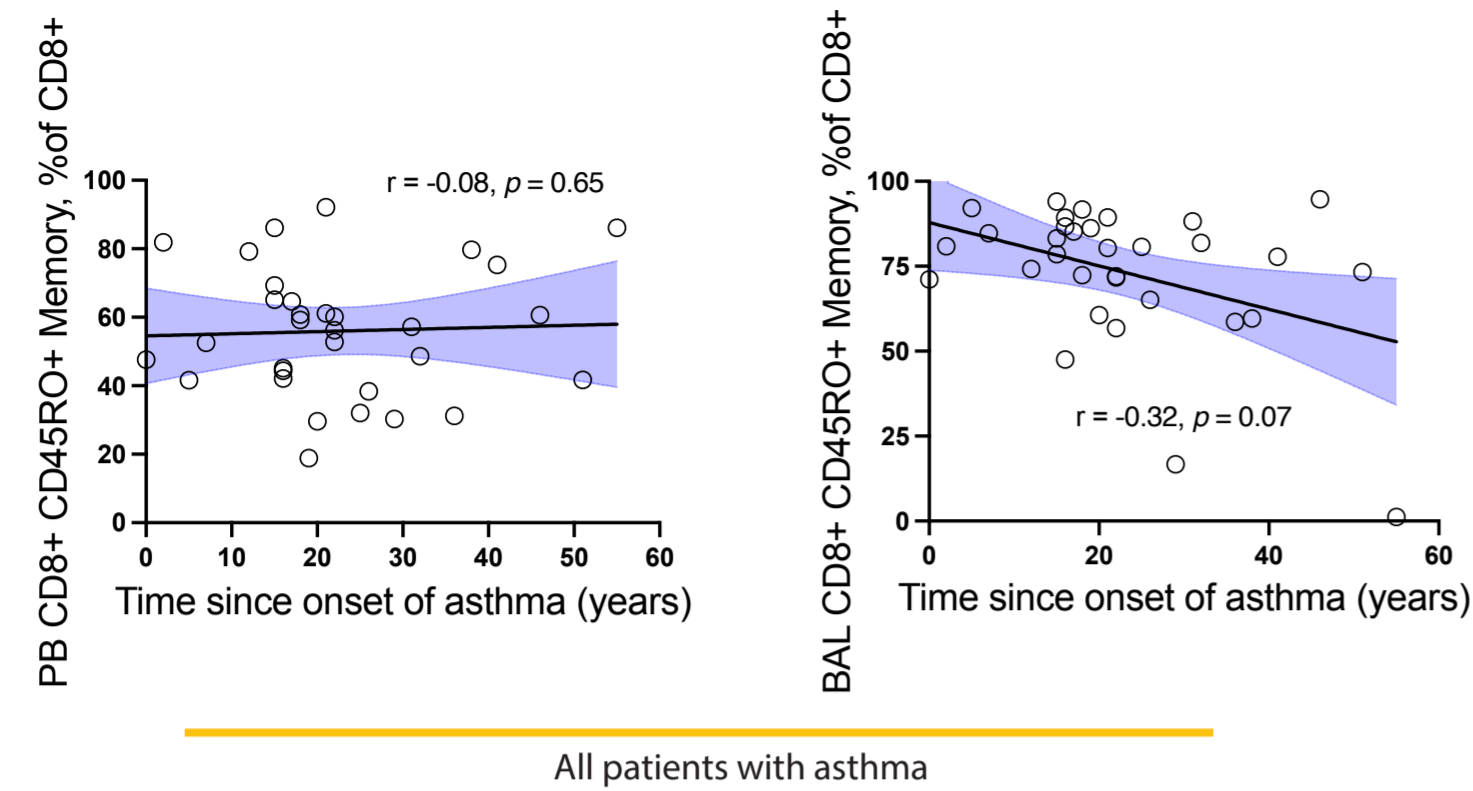

B

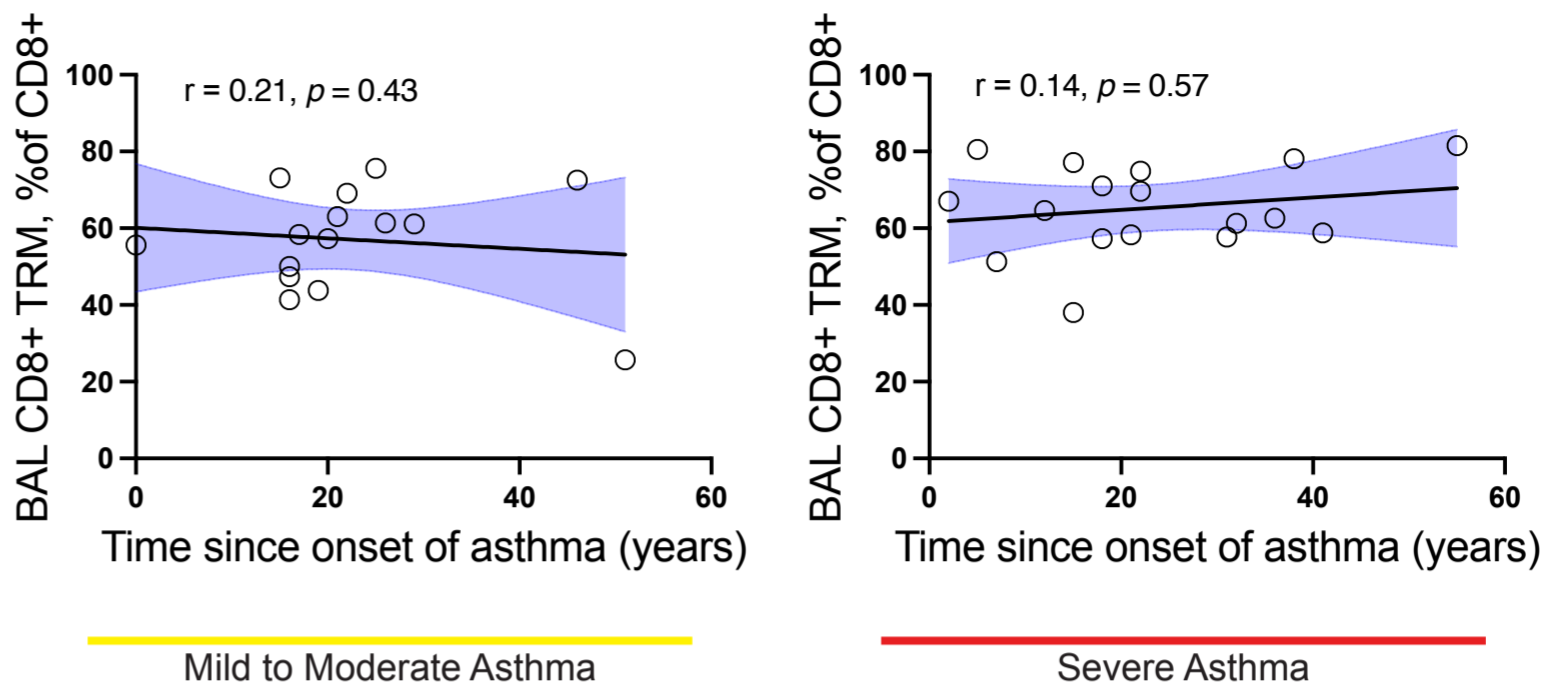

C

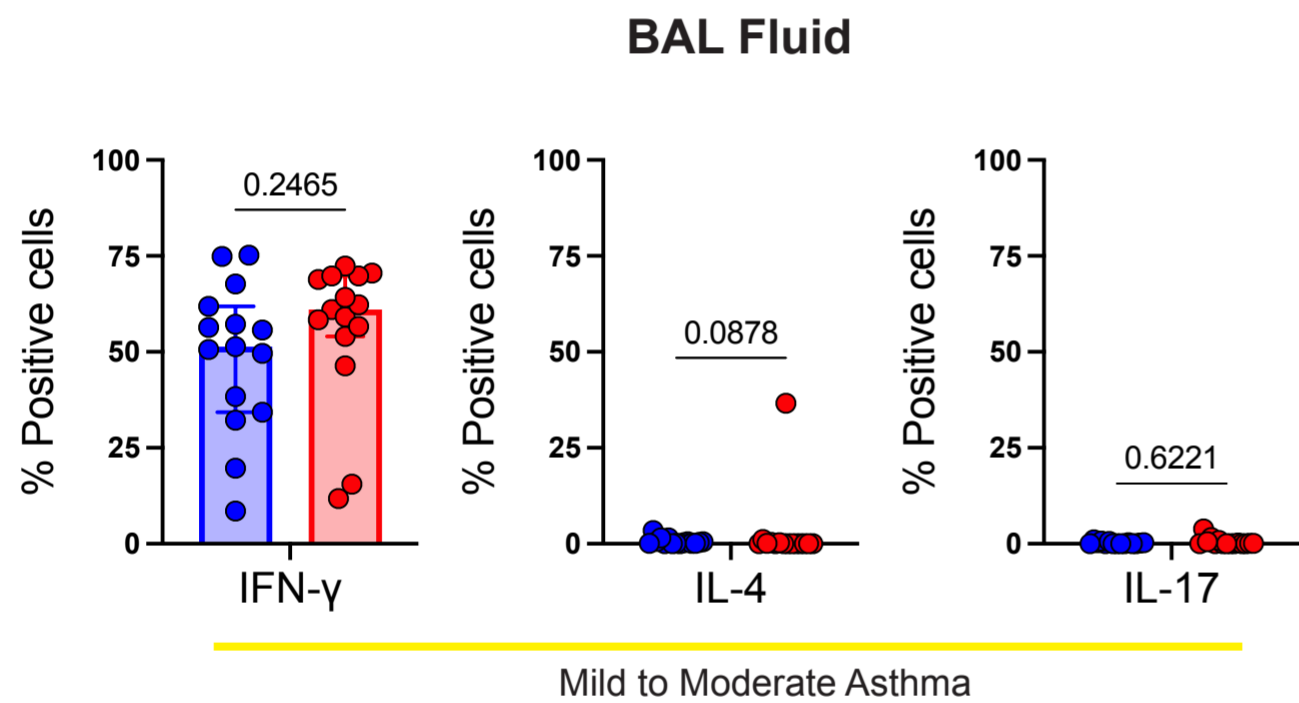

D

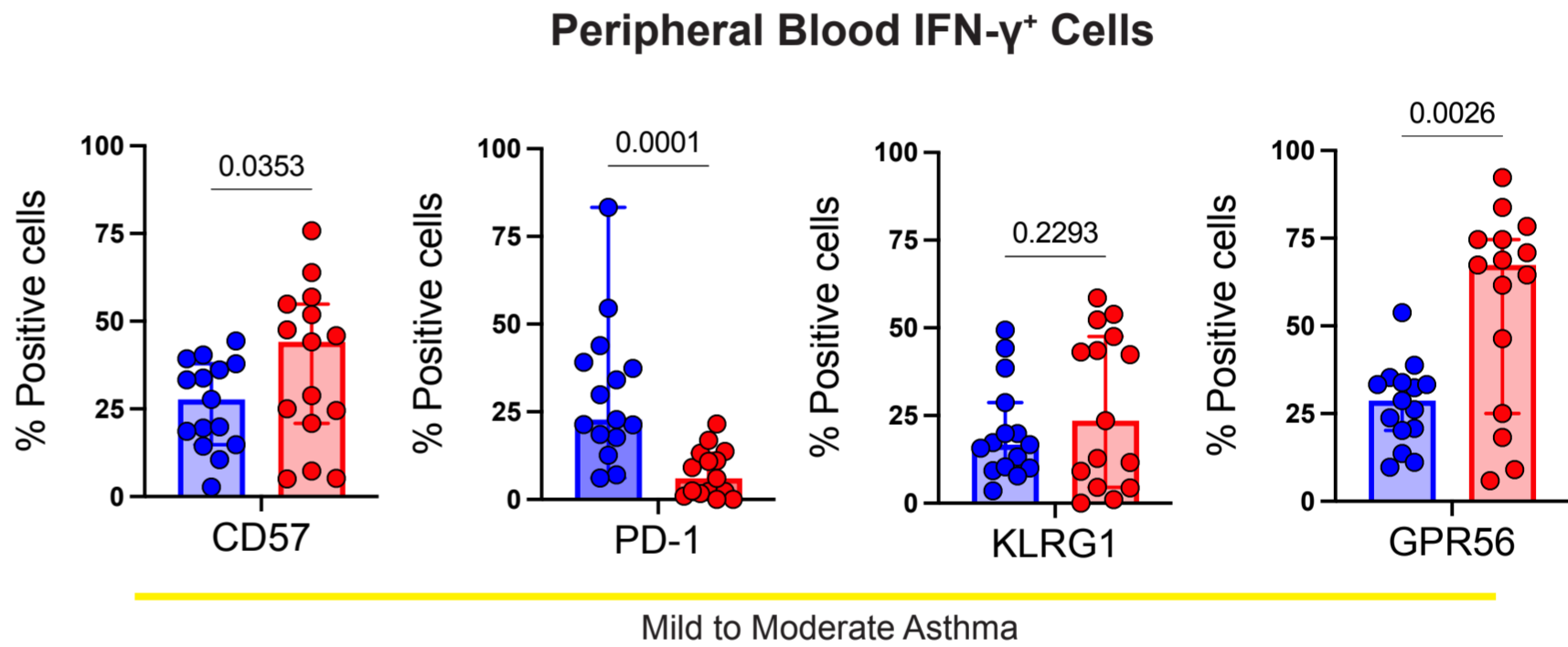

● CD8+ CD45RO+ Memory  
● CD8+ TEMRA

E

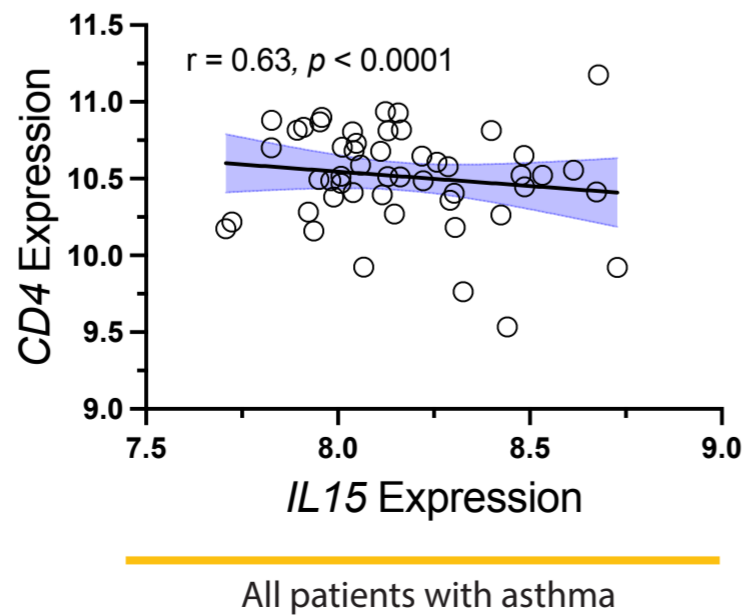

F

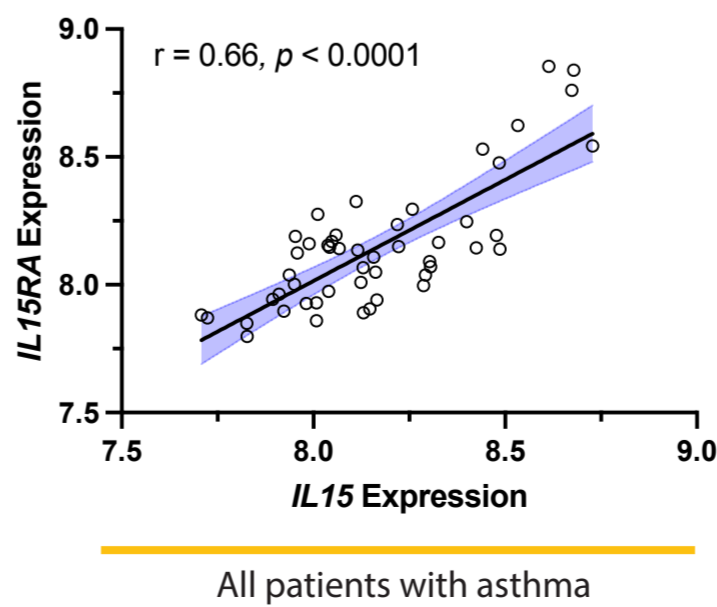

Supplement: Supplemental data [file jciinsight-10-185061-s029.pdf]
